# Distribution and bioavailability of mercury in the surface sediments of the Baltic Sea

**DOI:** 10.1007/s11356-021-13023-4

**Published:** 2021-03-06

**Authors:** Urszula Kwasigroch, Magdalena Bełdowska, Agnieszka Jędruch, Katarzyna Łukawska-Matuszewska

**Affiliations:** Institute of Oceanography, University of Gdańsk, Piłsudskiego 46, 81-378 Gdynia, Poland

**Keywords:** Mercury, Speciation, Surface sediments, Inflow events, Sea-dumped munition, Baltic Sea

## Abstract

The study aimed to determine the level of mercury (Hg) and its labile and stable forms in the surface sediments of the Baltic Sea. The work considers the impact of current and historical sources of Hg on sediment pollution, together with the influence of different environmental parameters, including water inflows from the North Sea. Surface sediments (top 5 cm) were collected in 2016–2017 at 91 stations located in different areas of the Baltic Sea, including Belt Sea, Arkona Basin, Bornholm Basin, Gdańsk Basin, West Gotland Basin, East Gotland Basin, and the Bothnian Sea. Besides, the particulate matter suspended in the surface and near-bottom water was also collected. The analysis of total Hg concentration and individual Hg forms in collected samples was carried out using a 5-step thermodesorption method. This method allows for the identification of three labile and thus biologically available, fractions of Hg, which are mercury halides, organic Hg, mercury oxide and sulphate. Two stable fractions, mercury sulphide and residual Hg, were also determined. The highest Hg concentrations, reaching 341 ng g^−1^, were measured in the highly industrialised Kiel Bay, which was additionally a munition dumping site during and after World War II. High Hg level, ranging from 228 to 255 ng g^−1^, was also recorded in the surface sediments of the Arkona Basin, which was a result of the cumulative effect of several factors, such as deposition of Hg-rich riverine matter, favourable hydrodynamic conditions and military activities in the past. The relatively elevated Hg concentrations, varying from 60 to 264 ng g^−1^, were found in the Gdańsk Basin, a region under strong anthropopressure and dominated by soft sediments. The sum of labile Hg in sediments was high and averaged 67% (with the domination of organic Hg compounds), which means that a large part of Hg can be released to the water column*.* It was found that the water inflows from the North Sea intensify the remobilisation of Hg and its transformation into bioavailable labile forms. As a consequence, the load of Hg introduced into the trophic chain can increase. Despite the significant reduction of Hg emission into the Baltic in the last decades, surface sediments can be an important secondary Hg source in the marine ecosystem. This is especially dangerous in the case of the western Baltic Sea.

**Supplementary Information:**

The online version contains supplementary material available at 10.1007/s11356-021-13023-4.

## Introduction

Owing to its high toxicity, mobility and stability, mercury (Hg) is considered one of the most dangerous elements found in the environment (UNEP 2013, 2019). Hg accumulates in living organisms and biomagnifies in the food chain. As a consequence of that, its concentrations in organisms, especially those occupying high trophic positions, are many times higher than those found in the surrounding environment (Jackson 1998; Fitzgerald et al. 2007). The toxicity of Hg depends on the form in which the element occurs: labile forms loosely bound to the matrix can be more easily transformed and absorbed by organisms, whereas stable forms are not bioavailable (Rice et al. 2014; Huang et al. 2020). The most toxic form of this metal is highly bioavailable methylmercury (MeHg), which is formed in the presence of bacteria, such as sulphate-reducing bacteria (SRB) or iron-reducing bacteria (IRB) in the process of methylation (Boeing 2000; Rodriguez Martín-Doimeadios et al. 2004; Yu et al. 2012). Even low levels of Hg in the body can lead to disruptions of important biochemical processes, irreversible disorders in the nervous system and brain functions (Axelrad et al. 2007). For that reason, the most vulnerable to the effects of Hg are organisms at the top of the trophic chain: predatory fish, mammals and birds, but also humans. Moreover, the metal possesses hepatotoxic, embryotoxic and mutagenic properties and may lead to cardiovascular disorders (Bose-O'Reilly et al. 2010; Roman et al. 2011). Hg also adversely affects organisms occupying lower trophic levels. In plants, Hg inhibits photosynthesis and reduces seed viability (Patra and Sharma 2000). In animals, the negative impact of Hg is notable already in invertebrates. Even small doses of Hg led to changes in their physiology, negatively influencing, e.g., food intake and digestion processes, respiration and metabolism, the reproduction and development of organisms. Hg also leads to behavioural changes, such as reduced locomotive activity, abnormal social behaviour or impaired defence mechanisms against predators and less effective hunting techniques (Krell et al. 2011; Weis 2014; Ackerman et al. 2016). Given the fact that the most common cause of Hg poisoning is the consumption of fish and seafood (Sunderland 2007; Rice et al. 2014; Mosa and Duffin 2017), the examination of Hg level and bioavailability in the marine environment is of particular importance.

**Fig. 1:**
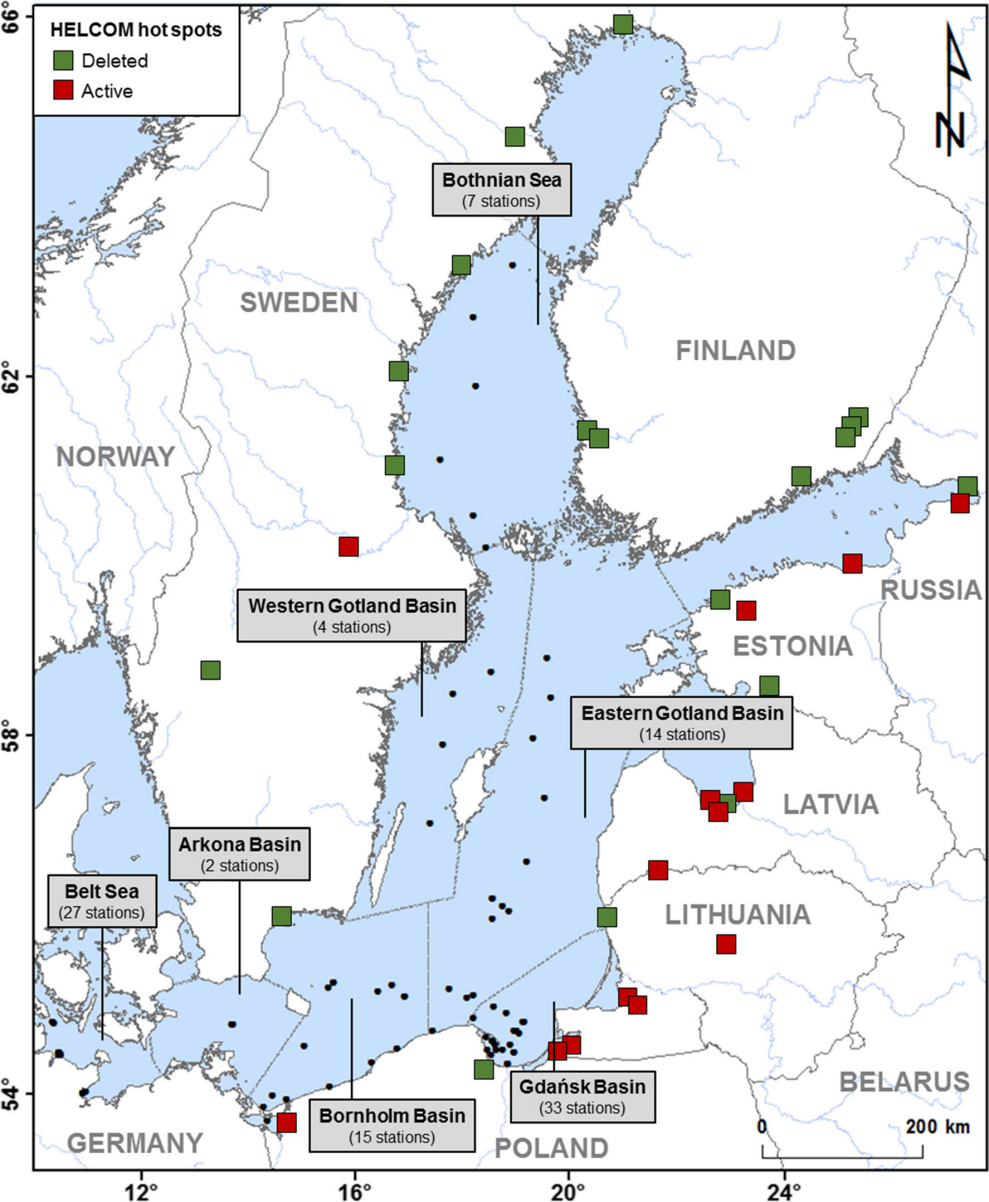
Map of the sub-basins of the Baltic Sea with the location of sampling stations in and the industrial hot spots in the Baltic Sea catchment area (HELCOM 2010)

The Baltic Sea is a semi-closed inland water basin exposed to strong human pressure from the surrounding industrialised and urbanised areas (Fig. 1). The water exchange with the Atlantic Ocean is limited and takes place mainly during episodic inflows of water from the North Sea through the Danish Straits. Many dangerous pollutants (Lilja et al. 2009; Chen et al. 2013; Staniszewska et al. 2015), including Hg, are introduced to the Baltic mainly via rivers, whose contribution in the Hg load is predominant, amounting to about 70%. The remaining 30% of the Hg load entering the basin comes from other sources, such as wet and dry atmospheric deposition and shore erosion (HELCOM 2010; Bełdowska et al. 2016). However, the atmospheric inflow of Hg is of particular importance in areas that are remote from anthropogenic sources. The transport of Hg (mainly in gaseous elemental form, Hg^0^) over long distances is possible due to the atmospheric residence time of Hg^0^, which is 6 to 12 months (Mason 2009). Besides, as a result of Hg^0^ reacting with halides that occur in oversea areas, Hg^0^ can be transformed into reactive gaseous mercury (RGM) (Hedgecock and Pirrone 2001). Hg is removed from the atmosphere with precipitation and through dry deposition, thus entering the seawater and can be easily transformed into bioavailable form (Hall et al. 2005; Zhang et al. 2009). Moreover, in highly urbanised areas such as the Baltic Sea, an important role is played by point sources such as coal and metal ore mines (located mainly in the southern part of the catchment area, in Germany and Poland), steel and metal industry (located in Estonia, Germany, Poland, Russia, and Sweden), wood and paper mills (located in the northern part of the Baltic region, in Finland and Sweden), dumpsites of ammunition and military waste, such as bombs and torpedoes (located mostly in the Bornholm Basin and the Gdańsk Basin) and ship and aircraft wrecks (Lepland et al. 2010; Leipe et al. 2013; HELCOM 2010, 2018a) (Fig. 1). Since the 1990s, a decrease in Hg concentration in the surface layer of the sediments has been noted. However, this trend is modified by inter-annual variability or the occurrence of extreme phenomena, such as floods. The impact of floods on the increase of Hg level in the surface sediments was visible, i.e., in the southern Baltic Sea during two floods in 1997 and 2010 (Saniewska et al. 2014; Jędruch et al. 2015). In highly polluted areas of the Baltic Sea, such as munition dumpsites, Hg level in sediments is still elevated (Bełdowski et al. 2016, 2019).

Sediments are an integral part of the marine environment and constitute a habitat and a source of nutrition for numerous organisms. Sedimentary organic matter is main diet component especially for surface detritus feeders and subsurface detritus feeders (burrowers), including mussels, gastropods, crustaceans and oligochaetes. It is also an important source of nutrition for consumers with flexible feeding strategy, especially in winter when availability of food is limited (Zaborska et al. 2018; Ziółkowska et al. 2018). In the coastal areas of the Baltic Sea, sedimentary organic matter is abundant with plankton or diatom detritus (Jędruch et al. 2017; Winogradow and Pempowiak 2018; Witak et al. 2020), while in the areas with the large benthic vegetation, the sediments also contained a lot of degraded vascular plants and macroalgal detritus (Jankowska et al. 2018; Ziółkowska et al. 2018). In the depositional areas of the Baltic Sea, such as Gdańsk Deep or Gotland Deep, the contribution of the autochthonous organic matter decreases in favour of terrestrial matter, especially in the deeper layers of the sediments (Szczepańska et al. 2012; Winogradow and Pempkowiak 2018). With the uptake of sedimentary organic matter by benthic meio- and macrofauna, the pollutants accumulated in it are introduced into the food chain. Hg found in sediments poses a great threat to the environment as its concentrations in organisms with a high position in the food chain are many times higher than in the organisms at low trophic level (Jędruch et al. 2018).

It should be noted that under the influence of many biotic and abiotic processes that occur in bottom sediments (e.g., bioturbation, resuspension and diffusion), Hg contained in them can be released to the near-bottom water and pore water (Pempkowiak et al. 2002; Sunderland et al. 2004; Fitzgerald et al. 2007; Bełdowski et al. 2009). To better identify the processes that determine the bioavailability of Hg in sediments, it is important to determine not only the total Hg concentrations but also the contribution of its forms. The level of Hg and its chemical form in the sediments depends on the origin of organic matter, for example, it is affected by the concentration and form of Hg in the organisms which sink to the bottom when they die, as well as in animal faeces or inorganic particles (Jędruch et al. 2017; Grajewska et al. 2020). The form of Hg in marine sediments is influenced by several physicochemical processes, such as adsorption onto clay minerals and organic matter, the formation of complexes with organic and inorganic ligands, precipitation and co-precipitation (mainly mercury sulphide (HgS)), oxidation and reduction reactions and the formation of the most dangerous metal-organic compounds (mainly MeHg) (Jackson 1998; Bełdowski and Pempkowiak 2007; Bełdowski et al. 2014). Salinity can also be an important factor conditioning Hg transformations on the water-sediment interface it can affect the methylation potential of Hg (Braaten et al. 2014; Chen et al. 2015; de Oliveira et al. 2015; Jonsson et al. 2014). Salinity can also impair the Hg adsorption onto clay minerals (Green-Ruiz 2009), thus affecting the release of Hg into the near-bottom water. In the Baltic Sea, salinity levels rise mainly during inflows of salinated and oxygenated water from the North Sea. These can also lead to a change in the reduction conditions in the sediment from anaerobic to aerobic and consequently leading to the transformation of stable HgS into bioavailable sulphate form, which can undergo methylation (Adriano 2001; Rodriguez Martín-Doimeadios et al. 2004).

The main aim of this study was to determine the level of total Hg concentration and the proportions of its labile and stable forms in the surface sediments of the Baltic Sea, taking into account the differences resulting from diverse anthropopressure and varied environmental conditions (including inflows from the North Sea).

## Materials and methods

### Study area

The study was conducted in the Baltic Sea (Fig. 1), which is an inland sea with limited water exchange with the North Sea and Atlantic Ocean. The Baltic Sea is characterised by reduced salinity and is one of the biggest brackish seas in the world. The salinity gradient stretches from the Danish Straits (15-20 PSU) located in the western part of the sea, across the central part—Baltic Proper (7-8 PSU)—to the Bothnian Bay (1-3 PSU) located in the north (HELCOM 2018b). The low salinity of the Baltic Sea is the result of the limited inflow of salinated ocean waters together with a large inflow of fresh water from rivers, located mainly in the southern and eastern parts of the catchment. Salinity of the Baltic Sea is affected by episodic inflows of salinated water from the North Sea, such as strong inflow which occurred in 2014–2016 (Mohrholz et al. 2015; Naumann et al. 2018). The inflows affect not only the salinity of the Baltic but also the aerobic situation in the water. In the Baltic Sea, there are many regions affected by oxygen depletion: hypoxia (O_2_ < 2 ml L^−1^) and anoxia (O_2_ ≤ 0 ml L^−1^) (Hansson et al. 2018). Hypoxia kills bottom-living organisms altering benthic faunal communities and impairing fish habitats (Conley et al. 2009). Moreover, hypoxic and anoxic conditions play and important role in the remobilisation of Hg from the sediment-water interface. Benthic Hg fluxes, including release of MeHg from sediments, under anoxic conditions are higher than in the normoxic (O_2_ > 2 ml L^−1^) environment (Emili et al. 2011). Consequently, the presence of anoxic areas could increase Hg concentrations in water and biota of the Baltic Sea. The hypoxia or anoxia results from the severe eutrophication of the Baltic Sea combined with the low ventilation of the Baltic Sea’s deep waters layers (Soerensen et al. 2016). The eutrophication is mainly associated with the anthropogenic inflow of biogenic substances to the sea from the catchment area, especially given the fact that its significant part is human-impacted with a large share of arable lands, urban and industrial centres (HELCOM 2010) (Fig. 1). The inflow of oxygenated waters from the North Sea improves the aerobic situation in the near-bottom zone. That, however, also depends on how strong the water column stratification is. In the case of heavy salinity stratification in the Baltic Proper region (which occurred during the 2000 inflow), the inflow may not be strong enough to penetrate the halocline and ventilate the deep water layer. Long-term retention of water under the halocline together with trapped organic particles leads to the intensification of hypoxia (Conley et al. 2009). Oxygen depletion in near-bottom waters creates anoxic conditions in sediments, which in turn can affect the occurrence of H_2_S in sediments and interstitial waters.

The research was conducted in 7 sub-basins of the Baltic Sea: Belt Sea, Arkona Basin, Bornholm Basin, Gdańsk Basin, West Gotland Basin, East Gotland Basin and the Bothnian Sea. The Belt Sea is the westernmost part of the Baltic Sea (Fig. 1). It is connected to the North Sea by a series of bays and straits, enabling water exchange between these basins and is a highly urbanised and industrialised region. The Arkona Basin, located between the Danish Belt Sea and the Baltic Proper (Fig. 1), is strongly influenced by water exchange between the North and the Baltic Sea and the inflow of contaminants derived from a large catchment area (Leipe et al. 2013). The Bornholm Basin is connected to the Arkona Basin by the Bornholm Gat, a large-scale submarine channel (Fig. A1). The Bornholm Basin receives laterally transported sediment material from the West and partly also from the South via the river Odra (Edelvang et al. 2002; Christoffersen et al. 2007; Leipe et al. 2013). The Gdańsk Basin provides an excellent example of the direct deposition area of river discharge (Bełdowski and Pempkowiak 2007; Jędruch et al. 2015). The basin is located just outside the outlet of the river Vistula, one of the largest rivers discharging into the Baltic Sea (Fig. 1), and due to shape and relatively large depth (Fig. A1), the sediments of the Gdańsk Basin are prone to accumulation of pollutants (Leipe et al. 2013; Saniewska et al. 2014; Jędruch et al. 2015). The Gotland Basin is characterised by a permanent pycnocline, which results in the absence of vertical mixing and oxygen transport to deeper water layers. This effects the presence of the largest anoxic zones in the Baltic Sea region (Leppäranta and Myberg 2008; Prien and Schulz-Bull 2016). In addition, the Eastern Gotland Basin, due to its shape and considerable depth (Fig. A1), is the place of final deposition of Hg introduced into the Baltic Sea from various sources located on land, as well as by atmospheric deposition (Leipe et al. 2013). The Bothnian Sea is located in the southern part of the Bothnian Bay, which is the northernmost part of the Baltic Sea (Fig. 1). The Bothnian Sea is separated from the Baltic Proper by the Åland Islands, which consequently limits the exchange of water in the reservoir and results in different hydrodynamic conditions (Raateoja 2013).

### Sample collection and analysis

Samples for analysis were collected during research cruises aboard the RV Oceania in the years 2016–2017. Samples of surface sediment (5 cm top layer) were collected with a van Veen grab sampler, box-corer or GEMAX corer at 91 stations located in various areas of the Baltic (Fig. 1, Table A1). The sediments were placed in polyethylene containers, previously cleaned with 4 M dilute HNO_3_ and rinsed with deionised water. The redox potential (Eh) of each sediment sample was measured immediately after collection using a portable meter (ProfiLine Multi 3320, WTW). An additional part of the material was put aside to analyse the grain sizes and organic matter content. Suspended matter (SPM) in seawater was collected from the surface layer (1 m depth) and the near-bottom layer (1 m above the seabed). Water samples were collected using a Niskin bottle and poured directly into 1 L^−1^ dark borosilicate bottles, previously cleaned with 4 M dilute HNO_3_, rinsed with deionised water and ignited (300 °C, 6 h). In order to obtain SPM, the water samples were filtered through fired (500 °C, 6 h) and pre-weighed Whatman GF/F glass microfiber filters (pore size 0.7 μm, diameter 47 mm) under a laminar flow cabinet. During filtration, blanks consisting of 1 L^−1^ of Milli-Q water were also prepared. Collected sediments and filters with SPM were frozen at − 20 °C and then freeze-dried (Alpha 1-4 LDplus, Martin Christ) (Eljarrat 2012). Before analysis, the sediment samples were additionally homogenised in a ball mill (8000D, Mixer/Mill, SPEX) with a tungsten vessel.

The analyses of total mercury (Hg_TOT_) concentration and Hg speciation in the sediments and SPM samples were performed using a direct mercury analyser (DMA-80, Milestone). Hg fractionation was carried out using the thermodesorption method described by Saniewska and Bełdowska (2017), further modified by Bełdowska et al. (2018) and Jędruch et al. (2018). Sediment samples were weighed in triplicate (sample weight 0.1 g) onto quartz vessels, which had previously been etched in 4 M HNO_3_ and ignited 800 °C, 1 min. Then, the sediment was heated at the following temperatures: 175 °C, 225 °C, 325 °C, 475 °C and 750 °C (a procedure which made it possible to separate labile and stable Hg fractions). The first labile fraction, released at 175 °C, is Hg compounds loosely bound with halides (e.g., HgBr_2_ and HgCl_2_), which are mainly adsorbed on the sediment surface (Hg_ads1_) (Bełdowska et al. 2018). This fraction also contains a gaseous Hg, which is released at the temperature of 100–125 °C and usually represents a small proportion in total Hg in the sediments (Coufalík et al. 2012; Bełdowska et al. 2018). The next fraction to decompose, at 225 °C, is organically bound Hg, including one of the most toxic forms such as monomethyl mercury (MeHg) (Hg_abs_). However, based on the results of the previous studies conducted by authors, the proportion of MeHg in the Hg_abs_ varied widely from 50% to almost 100% depending on the matrix used (Saniewska and Bełdowska 2017; Jędruch et al. 2018). At the next temperature level (325 °C), mercury sulphide (HgS) is released. This is the stable Hg form that is most commonly found in the environment. At 475 °C, it was possible to observe the decomposition of semi-labile, adsorbed Hg forms such as HgO and HgSO_4_ (Hg_ads2_). At the highest temperature of 750 °C, Hg compounds are decomposed in a form bound with the residual fraction, that is not available to the environment (Hg_res_) (Bełdowska et al. 2018). The application of the 5-step thermodesorption method enabled the identification of 5 groups of Hg compounds with similar properties, including comparable binding to the sediment particles and, moreover, their bioavailability. However, Hg bioavailability is defined differently between studies and is usually used non-specifically and interchanged with other more general or fundamentally different terms, e.g., solubility, lability, availability and reactivity (Semple et al. 2004; Huang et al. 2020). Here, we recognise ‘bioavailable Hg’ as the pool of labile Hg forms which can migrate to the solution phase in response to changes in the physicochemical conditions (e.g., pH and Eh) and therefore are potentially mobile and available for uptake or assimilation by organisms. This pool incudes three labile forms of Hg (adsorbed Hg, mainly bound with halides (Hg_ads1_), Hg absorbed in organic matter (Hg_abs_), adsorbed Hg oxide and Hg sulphate (Hg_ads2_)). The other two stable forms (HgS and residual Hg) are biologically unavailable. HgS can, however, be transformed into an organic form with the participation of sulphate-reducing bacteria and by fungi (Rodriguez Martín-Doimeadios et al. 2004), so is therefore only temporarily beyond the reach of organisms.

The accuracy of the method used was verified by comparing the sum of Hg concentrations measured by the 5-step fractionation method with the result of the Hg_TOT_ analysis. The accuracy of the Hg forms analysis averaged 94%, with a standard deviation not exceeding 5%. Accuracy and precision of the method were verified by the analysis of certified reference materials (soil NCS DC 87103—Hg_TOT_ 17 ng g^−1^ and marine sediment GBW 07314 - Hg_TOT_ 43 ng g^−1^). The analysis of reference materials was carried out in three replicates, for which the recovery was found to be at levels of 96 and 98%, respectively. The limit of detection (LOD) calculated according to the IUPAC (1997) equation:

$$ \mathrm{LOD}={\mathrm{x}}_{\mathrm{blank}}+k\ {\mathrm{SD}}_{\mathrm{blank}}, $$

where *x*_blank_ is the arithmetic mean of 10 blank (analyte-free samples) measurements, SD_blank_ is the blank standard deviation, and *k* is a numerical factor. The value chosen for *k* was 3 as recommended by IUPAC (1997). The LOD was calculated for each of the five fractionation temperatures. In any case, the calculated value did not exceed 0.01 ng of Hg.

Organic matter content, expressed as loss on ignition (LOI), was determined by combusting a sediment sample at 550 °C for 6 h (Ciborowski 2010). A granulometric analysis was carried out to determine the contribution of individual fractions in the collected sediments. Sediment samples were sieved (for 10 min) through 6 sieves with the following mesh sizes: 2, 1, 0.5, 0.25, 0.125 and 0.063 mm. Sediments with a grain size less than 0.063 mm (silt and clay) were classed as fine sediment fraction (FSF). The size fractions were determined using Udden’s classification (1914), further modified by Wentworth (1922). The sediment type was determined by the ratio of grain size fractions in the sediments, using the Shepard (1954) classification. The types of bottom, including sand, soft (silt, clay, mud) and till (unsorted sediment deposited by a glacier), were distinguished on the basis of the classification proposed by Winterhalter et al. (1981) (Fig. A2).

### Data processing

All of the obtained concentrations are presented on the basis of sample dry weight (dw). Statistical analysis was carried out using the STATISTICA 12 (StatSoft) software. The obtained results of Hg_TOT_ concentration and the proportion of Hg forms were characterised by a nonparametric data distribution (Shapiro-Wilk test, *p* = 0.000). In order to determine the significance of differences, the non-parametric U Mann-Whitney test, Kruskal-Wallis test and multiple comparison post-hoc Dunn’s test were used. The hypotheses were tested at a statistical significance level of *p* < 0.05. The correlations between the analysed variables were determined on the basis of Spearman’s correlation coefficients. The maps of the study area and the spatial interpolation (inverse distance weighting (IDW) method by Burrough et al. (2015)) of Hg concentrations were created using the ArcMap 10.4.1 (ESRI) with the geographic coordinate system chosen for data presentation WGS1984. Part of the basic spatial data was provided courtesy of the GIS Centre, University of Gdańsk (www.cgis.oig.ug.edu.pl).

## Results and discussion

### Concentration and forms of Hg in the Baltic Sea

#### Surface sediments

The concentrations of total mercury (Hg_TOT_) in surface sediments collected from the Baltic Sea ranged widely from 1.2 to 340.8 ng g^−1^ (median 57.8 ng g^−1^). These results were similar to those measured in the surface sediments of the Baltic Sea in 1993 by Perttilä et al. (2003), which had ranged from 13 to 406 ng g^−1^, as well as those observed in 2001–2008 by HELCOM (2010), which had ranged from 40 to 300 ng g^−1^. This may indicate that despite the systematic reduction of the Hg emission to the environment (KOBIZE 2019; Jędruch et al. 2021) and Hg inflow to the Baltic since the beginning of the 1990s (HELCOM 2009; EMEP 2018), the Hg concentration in surface sediments has not decreased significantly.

Hg_TOT_ concentrations in Baltic sediments were many times lower than the values measured in heavily polluted areas, such as northern Adriatic near the Idria mercury mining district in Slovenia, and about 2–3 times higher than measured in the sediments of the Arctic seas considered to be non-contaminated areas (Table 1). A comparison of the obtained results with the international threshold values for Hg in sediment showed that Baltic Sea sediments does not seem to be under high environmental risk, as far as Hg contamination is concerned. Hg_TOT_ concentration in 63% of the analysed sediment samples did not exceed the 100 ng g^−1^, which is defined as Background Assessment Concentration (BAC) and was set by the Convention for the Protection of the Marine Environment of the North-East Atlantic (OSPAR Convention) (OSPAR 2009). This value was developed for testing whether measured Hg concentrations are near background levels for naturally occurring substances and close to zero for man-made substances. What is more, over 81% of the obtained results on Hg_TOT_ in surface sediment were lower than 150 ng g^−1^, which is a value used by the United States National Oceanic and Atmospheric Administration (NOAA) for assessing the ecological significance of contaminant concentrations in sediment, to protect against the potential for adverse biological effects on organisms (NOAA 1999). This level is described as Effects Range-Low (ERL) and represents the Hg concentration in sediments below which no adverse effect on the marine organisms is observed.

**Table 1:** Comparison of the total mercury (Hg_TOT_) concentration (ng g^−1^) in the surface layer of marine sediments from various areas of the world

Region	Sampling period	Mean	Median	Range	Reference
Aegean Sea, Nemrut Bay	2005	5 750		1 700–9 600	Esen et al. 2008
Adriatic Sea, Gulf of Trieste	1995–1996	5 240	4 450	100–23 300	Covelli et al. 2001
Ionian Sea		2 770		360–7 730	Spada et al. 2012
Patos Lagoon, Brazil		2 390		20–17 840	Mirlean et al. 2003
Adriatic Sea, Marano-Grado Lagoon				680–9 950	Acquavita et al. 2012
Red Sea coast, Saudi Arabia	2018	1 830		680–3 800	Kahal et al. 2018
Adriatic Sea, Venice Lagoon	2005			209–1 144	Han et al. 2007
Mediterranean Sea, Haifa Bay	2007	255		79–347	Shoham-Frider et al. 2012
Bohai Sea, Laizhou Bay	2012	210		10–1 200	Zhuang and Gao 2015
San Francisco Bay	2001–2002	170		9–560	Lu et al. 2005
Sado Estuary, Portugal	2006		130	3–540	Lillebø et al. 2011
North Sea, Jade Bay	2007	103		8–243	Jin et al. 2012
Baltic Sea	2016–2017	84	58	1–341	This study
South China Sea	2000–2002	54	36	2–201	Shi et al. 2010
Chesapeake Bay	1995			80–180	Mason et al. 1999
Beaufort Sea	1999–2001	41	40	3–97	Trefry et al. 2003
Chukchi Sea	2009–2010	31	32	5–55	Fox et al. 2014
Kongsfjorden, Svalbard	2009	24		9–87	Liu et al. 2015
White Sea	2004	23		6–95	Fedorov et al. 2019
Persian Gulf	2016	22		8–34	Hassan et al. 2019

**Fig. 2:**
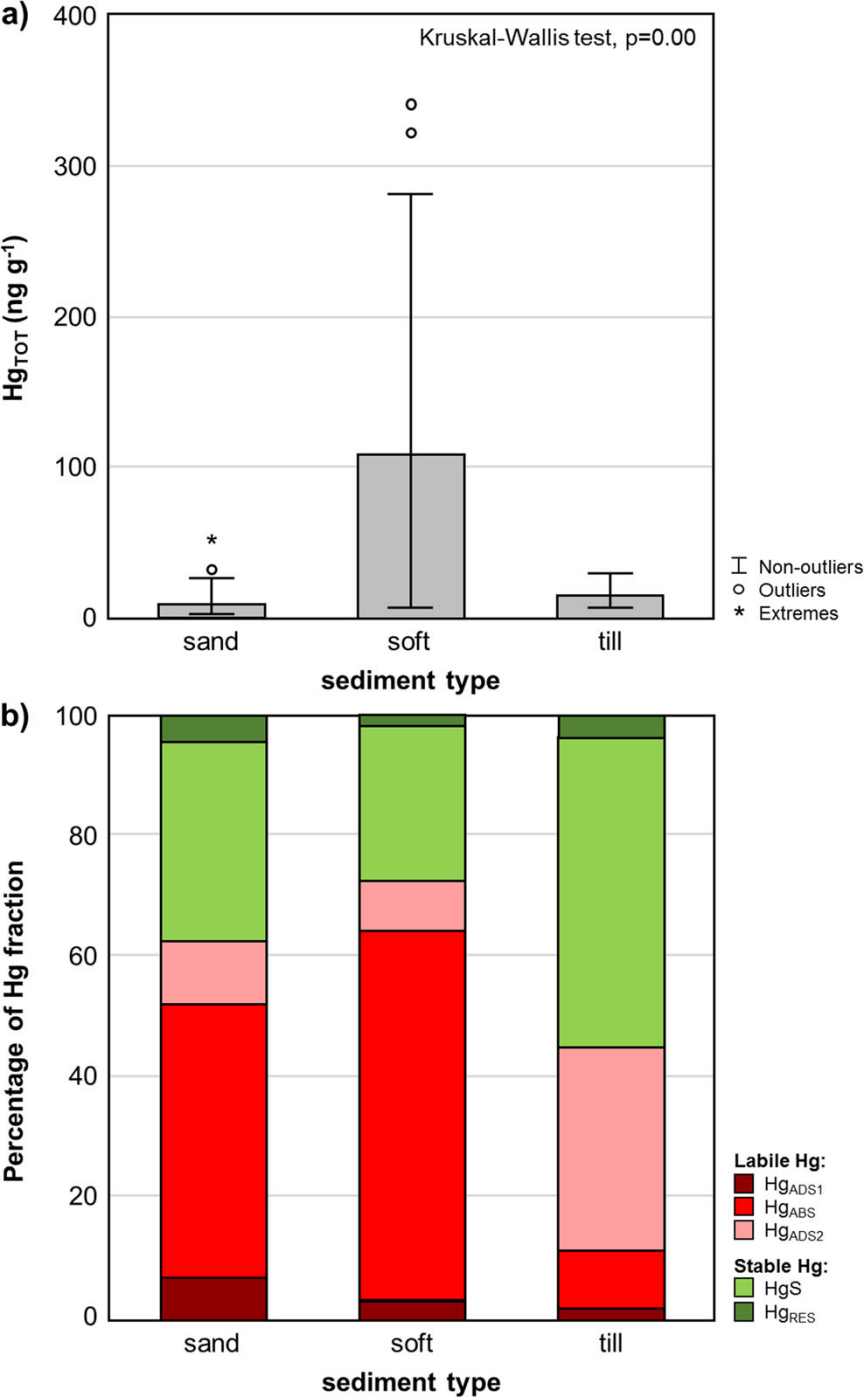
Concentration of the total mercury (Hg_TOT_) **a** and the contribution of labile and stable mercury fractions **b** in the different types of surface sediments from the Baltic Sea

Hg_TOT_ concentration in surface sediments was subject to statistically significant variability depending on the type of sediment (Kruskal-Wallis test, *p* = 0.000) (Fig. 2a). The lowest values in the range from 1.2 to 51.9 ng g^−1^ (median 8.7 ng g^−1^) were measured in sandy sediments. The Hg_TOT_ concentrations in till sediments were slightly higher, ranging from 7.5 to 30.5 ng g^−1^ (median 15.7 ng g^−1^) and did not differ statistically significantly from the values recorded in the sands (Dunn’s test, *p* = 0.356). The highest Hg_TOT_ concentrations were found in soft sediments and ranged from 8.4 to 340.8 ng g^−1^ (median 109.7 ng g^−1^ ). These values were statistically significantly different from those obtained in sandy sediments (Dunn’s test, *p* = 0.000) and in till sediments (Dunn’s test, *p* = 0.007) (Fig. 2a). The differences observed between the particular types of sediment were associated primarily with sediment parameters, such as FSF and LOI. This was confirmed by strong statistically significant correlations of Hg_TOT_ with both FSF (*R* Spearman = 0.87, *p* = 0.000) and LOI (*R* Spearman = 0.71, *p* = 0.000) (Table A3). The FSF content in the sediment is an important factor that determines the affinity of sediment particles with metal ions, including Hg. This is mainly due to an increase in the specific surface area of the sediment along with the decrease in grain size. Most of the processes responsible for metal sorption are surface reactions (Warren and Zimmerman 1994; Bengtsson and Picado 2008). FSF can be the sole factor controlling Hg concentration in sediments with low organic matter content and high SPM concentration, as observed, i.e., in Arctic Fiords (Jiang et al. 2011; Bełdowski et al. 2015). In contrast, organic matter has a strong capacity to bind and complex Hg (Pempkowiak 1997; Bełdowski and Pempkowiak 2003). Moreover, elevated Hg concentration in the sedimentary organic matter results from the fact that it consists largely of dead and decomposing plants and animals that have accumulated Hg in their cells and tissues. However, in the depositional areas of the Baltic Sea, the contribution of fresh autochthonic organic matter is low as in the course of early sediment diagenesis it undergoes further mineralisation after deposition (Winogradow and Pempkowiak 2018). A statistically significant negative correlation was found between Hg_TOT_ and the redox potential (*R* Spearman = -0.64, *p* = 0.000) (Table A3). This means that Hg_TOT_ concentrations in surface sediments increased when the oxygen conditions deteriorated. Despite the fact that both oxidised and reduced sediments can accumulate Hg, the hypoxic and anoxic conditions, together with the appearing of H_2_S, facilitate the accumulation of Hg in sediments (Ullrich et al. 2001; Zhu et al. 2018). Improvement in aerobic conditions can decrease concentration of H_2_S in the surface sediment and consequently less binding of Hg with sulphide in the sediment. Oxygenation of an anoxic sediment leads to the appearance of Hg species in the water column and the potential bioaccumulation by organisms. However, as shown by Pakhomova et al. (2018), any shifts in redox conditions in bottom water and upper sediment layer lead to the release of Hg species into the water column. The negative dependence between Hg concentration and redox potential was partly related to the station depth, which is confirmed by a statistically significant negative correlation between redox potential and depth (*R* Spearman = − 0.48, *p* = 0.003) (Table A3). The depth of the station was also an important factor determining the diversity and distribution of bottom sediments. In deep-water regions, the dominance of fine-grained sediments rich in organic matter was confirmed, which is confirmed by a statistically significant correlation between station depth and the presence of FSF (*R* Spearman = 0.48, *p* = 0.000) and organic matter (LOI) (*R* Spearman = 0.38, *p* = 0.002) (Table A3) in the sediments. There was also a correlation between the redox potential and the FSF in surface sediments (*R* Spearman = − 0.50, *p* = 0.001), as well as the redox potential and LOI (*R* Spearman = − 0.81, *p* = 0.000). This results from the fact that fine-grained sediments with a high proportion of organic matter and deposited at large depths are characterised by poorer oxygenation compared to the coarse-grained sediments located at shallow depths. The LOI dependence on FSF is additionally confirmed by a statistically significant correlation between the contribution of these parameters in the analysed sediments (*R* Spearman = 0.76, *p* = 0.000) (Table A3). Similar correlations in surface sediments of the Baltic Sea were noted, among others, by Bełdowski and Pempkowiak (2003), Jędruch et al. (2015, 2018), Kwasigroch et al. (2018) and Jędruch and Bełdowska (2020).

The proportions of Hg varied greatly among the determined three labile forms (adsorbed Hg, mainly bound with halides (Hg_ads1_), Hg absorbed in organic matter (Hg_abs_), adsorbed Hg oxide and Hg sulphate (Hg_ads2_)) and two stable forms (HgS and residual Hg) in the surface sediments of the Baltic Sea. The contribution of the Hg_ads1_ fraction ranged from 0.6 to 14.7% (median 2.9%), Hg_abs_ from 7.5 to 95.7% (median 52.0%), Hg_ads2_ from 0.9 to 37.1% (median 8.8%), HgS from 0.8 to 69.8% (median 30.9%) and Hg_res_ from 0.1 to 22.7% (median 1.3%). The total proportion of labile forms ranged from 28.9 to 99.0% (median 66.5%). As was the case with Hg_TOT_ concentration, the contribution of Hg forms in sediments depended on the type of sediment (Fig. 2b). Sandy sediment was dominated by the labile Hg_abs_ fraction, the proportion of which ranged from 7.5 to 70.0% (median 47.0%). Soft sediments were similar, with the contribution of this form ranging from 11.0 to 95.7% (median 60.9%). The case of till sediments was different: here, the predominant form was stable HgS which ranged from 47.0 to 57.7% (median 51.3%). A characteristic feature of till sediments was also the increased proportion of Hg_ads2_ (28.6–36.6%) compared to sand and soft sediments (Fig. 2b). This may be related to the different mineral composition of these deposits, in which higher iron concentrations were found than in other regions, as well as to their higher oxygenation. The contribution of Hg_abs_ fraction grew with an increase in Hg_TOT_ (*R* Spearman = 0.49, *p* = 0.000) (Table A3). A positive correlation between the concentration of Hg bound to the organic ligands and Hg_TOT_ in the Baltic sediments was also found in the study of Bełdowski et al. (2014). The proportion of Hg_abs_ fraction also increased with a drop in Eh (*R* Spearman = − 0.39, *p* = 0.004), a fact which may be related to Hg transformations occurring in sediments under anaerobic conditions due to the multiplication of bacteria (including sulphur and methane bacteria) (Ullrich et al. 2001; Zhu et al. 2018). These conditions were conducive to Hg methylation (Marvin-Dipasquale and Agee 2003), which in turn favoured an increase in the Hg_abs_ fraction.

In the case of Hg_abs_, a positive dependence was found between the contribution of the form and the redox potential (*R* Spearman = 0.50, *p* = 0.000) (Table A3). This indicates a high proportion of this fraction in well oxygenated sediments, which is also confirmed by the negative correlation of Hg_ads1_ with depth (*R* Spearman = − 0.55, *p* = 0.000), bearing in mind that shallow sediments were characterised by higher Eh values, as evidenced by the correlation between depth and Eh (*R* Spearman = − 0.48, *p* = 0.003). A higher Hg_abs_ contribution in shallow and oxic sediments of the Baltic Sea is probably associated with the presence of the fresh organic matter, both autochthonous and that introduced with surface run-off, in the coastal sediments, rather than with the methylation of Hg in sediments (Schartup et al. 2013; Pakhomova et al. 2018). The contribution of the third labile fraction, Hg_ads2_, increased as the Hg_abs_ proportion decreased (*R* Spearman = − 0.80, *p* = 0.000) and also when Eh increased (*R* Spearman = 0.41, *p* = 0.013). This is due to the fact that this fraction is constituted by Hg bounded with oxides and sulphates, which would become reduced under anaerobic conditions (Lasserre and Martin 1986). Taking into account the contribution of stable Hg forms, it is evident that it was significantly correlated with the proportion of the Hg_abs_ fraction. That is indicated by the inverse correlation between the share of labile Hg_abs_ and the proportions of stable forms: HgS (*R* Spearman = − 0.94, *p* = 0.000) and Hg_res_ *(R* Spearman = − 0.64, *p* = 0.000) (Table A3). This is related to the degradation of organic matter in sediments. Hg methylation potential changes during anaerobic processes in the seabed, leading to the formation of HgS (Bełdowski et al. 2009).

#### Suspended particulate matter

Hg_TOT_ concentrations in the suspended particulate matter (SPM) samples collected in the Baltic Sea region varied from 20.1 to 202.1 ng g^−1^ (median 56.3 ng g^−1^). A slightly higher median of Hg_TOT_ concentrations was recorded in surface water (median 64.9 ng g^−1^) than in near-bottom water (median 50.3 ng g^−1^). At the same time, there was no significant statistical difference between the Hg_TOT_ concentrations in SPM collected near the surface and at the bottom (*U* Mann-Withney test, *p* = 0.102). The concentration of Hg_TOT_ bound to SPM, in the Baltic waters, ranged from 0.1 to 2.1 ng L^−1^ (Table A2) and did not differ statistically (*U* Mann-Whitney test, *p* = 0.474) at particular depths: in both the subsurface and near-bottom water, the median was 0.3 ng L^−1^ (Table A2). The highest Hg_TOT_ concentrations in SPM (202.1 ng g^−1^) were measured in the Gdańsk Basin, in the near-bottom layer, at the station located south of the Hel Peninsula, in a sheltered part of the Gulf of Gdańsk. That indicates a significant Hg enrichment of SPM in near-bottom water, which most likely results from resuspension of fine-grained bottom sediments with a similar Hg_TOT_ concentration (263.6 ng g^−1^). For the sake of comparison, the concentration of Hg_TOT_ in SPM in surface water at this station was about 5 times lower (42.6 ng g^−1^) than in near-bottom water. That value was over 2 times higher than the average Hg_TOT_ concentration in SPM in the surface layer of water (16.2 ng g^−1^) and close to the maximum values recorded in this region in the preceding years (43.6 ng g^−1^). This may indicate an additional source of Hg to that of sediment remobilisation in the form of increased primary production during sampling (ciliate bloom season). Phytoplankton accumulates Hg; therefore, its blooms can lead to a several-fold increase of Hg_TOT_ in SPM (which phytoplankton is a part of) (Pickhardt and Fisher 2007; Hammerschmidt et al. 2013; Gosnell and Mason 2015; Bełdowska and Kobos 2016; Jędruch et al. 2017). The lowest Hg_TOT_ concentrations (20.1 ng g^−1^) in SPM were measured in near-bottom water in the Western Gotland Basin area at the station in Landsort Deep. This is probably related to the limited Hg inflow to the Baltic Sea in that region. The amount of surface runoff is of particular importance here—despite the fact that the fjards located west of the Landsort Deep (e.g., Himmerfjärden and Bråviken) introduce significant loads of matter into the basin, and most of this material undergoes sedimentation near the source and is not carried into deep-water regions (Kyryliuk and Kratzer 2019). The Hg inflow to the bottom regions of the Landsort Deep from other parts of the Baltic seabed is also limited—water masses contaminated with Hg-rich organic matter become diluted during horizontal transport (Meier et al. 2006). The inflow of Hg to the deep-water zone of the Landsort Deep is also hindered by a strong, permanent pycnocline, which prevents vertical mixing and with it the transport of Hg into the waters below (Prien and Schulz-Bull 2016). A decrease in the concentration of Hg in the SPM below this layer was also observed in other regions of the Baltic Sea—the Gdańsk Deep and the Bornholm Deep (Bełdowski et al. 2009).

The predominant form of Hg found in the analysed SPM samples from the Baltic Sea region was definitely H_ads1_ (Table A2), i.e., adsorbed Hg halides. Its contribution in SPM collected from surface water ranged from 61%, in SPM collected in the southern part of the Bornholm Basin, to 90% in the Belt Sea region, while the median was 84%. In the case of near-bottom water, Hg_ads1_ occurred in an even wider range: from 42% in SPM collected in the central part of the Gdańsk Basin to 91% in SPM in the sheltered part of the Gulf of Gdańsk. The median of Hg_ads1_ proportion in that case was 74%. The form with the second highest contribution was absorbed Hg, bound with organic matter (Hg_abs_). In surface SPM, its proportion ranged from 6% in the Eastern Gotland Basin area to 35% in the southern part of the Bornholm Basin (median 15%). In SPM collected from near-bottom water, the contribution of Hg_abs_ was similar and ranged from 8% in the central Gulf of Gdańsk region to 43.8% in the southern part of the Western Gotland Basin (median 20%). The proportion of stable HgS was very small, as is indicated by the results obtained for SPM in surface waters (median 1%) and in near-bottom water (median 2%). However, it is worth mentioning that there was a 17% HgS proportion at the station in the central Gulf of the Gdańsk area. In addition, the share of HgS was the only aspect that differed significantly in the surface water to near-bottom water ratio (*U*-Mann Whitney test, *p* = 0.008). None of the other cases was such a difference demonstrated. The contributions of labile Hg_ads2_ and stable Hg_res_ in SPM collected from all the regions were marginal (Table A2).

In SPM collected in the Baltic, the proportion of the dominant form Hg_ads1_ was higher than that measured in the SPM introduced into the Southern Baltic via rivers, such as the Reda (25–44%) or the Gizdepka (44–48%)—in these watercourses, the predominant form was Hg in organic bonds, whose proportion reached 61% (Saniewska et al. 2019). A similar proportion of Hg forms was also observed in the SPM in the Southern Baltic coastal zone, where the contribution of Hg_ads1_ ranged from 26 to 39%, while the proportion of Hg_abs_ was predominant and accounted for about 50% of Hg_TOT_ (Jędruch and Bełdowska 2020). The high proportion of Hg_abs_ in the riverine SPM and in the coastal zone of the sea can be associated with high primary production, which may include the presence of phytoplankton, in which the share of Hg in organic bonds is much higher (Le Faucher et al. 2014; Gosnell and Mason 2015; Bełdowska et al. 2018). Another possible reason is the outflow of Hg_abs_ from the soils in the catchment area (Saniewska et al. 2019). The increase in the contribution of Hg_ads1_ in the Baltic SPM may indicate mineralisation of organic matter as the distance from land-based Hg sources grows (Bradtke et al. 2005). This is supported by the lower contribution of Hg_abs_ in the sea SPM compared to the river SPM. It could also be an indication of the increased importance of atmospheric deposition driving the increased Hg adsorption to SPM (Bełdowska et al. 2018; Korejwo et al. 2020). The influence of the atmosphere on the proportion of Hg forms in SPM is confirmed by the higher proportion of the halide-related form in SPM in the surface water of the Baltic Sea (84%) compared to the near-bottom SPM (74%), but also the adsorption of ions dissolved in water, e.g., Hg chloride (Table A2).

### Spatial distribution of Hg concentrations

**Fig. 3:**
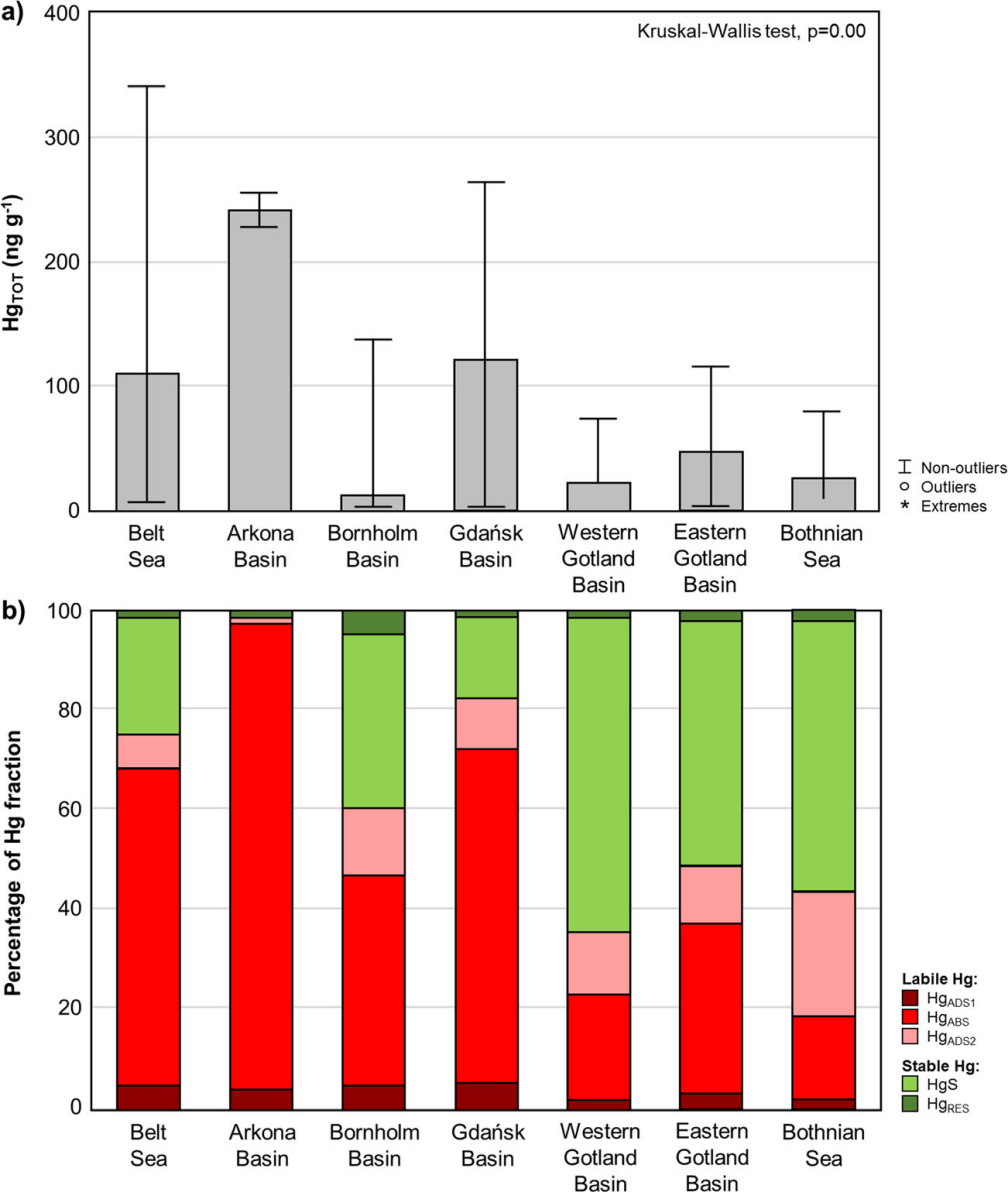
Concentration of the total mercury (Hg_TOT_) **a** and the contribution share of labile and stable mercury fractions **b** in the surface sediments from the different regions of the Baltic Sea

**Fig. 4:**
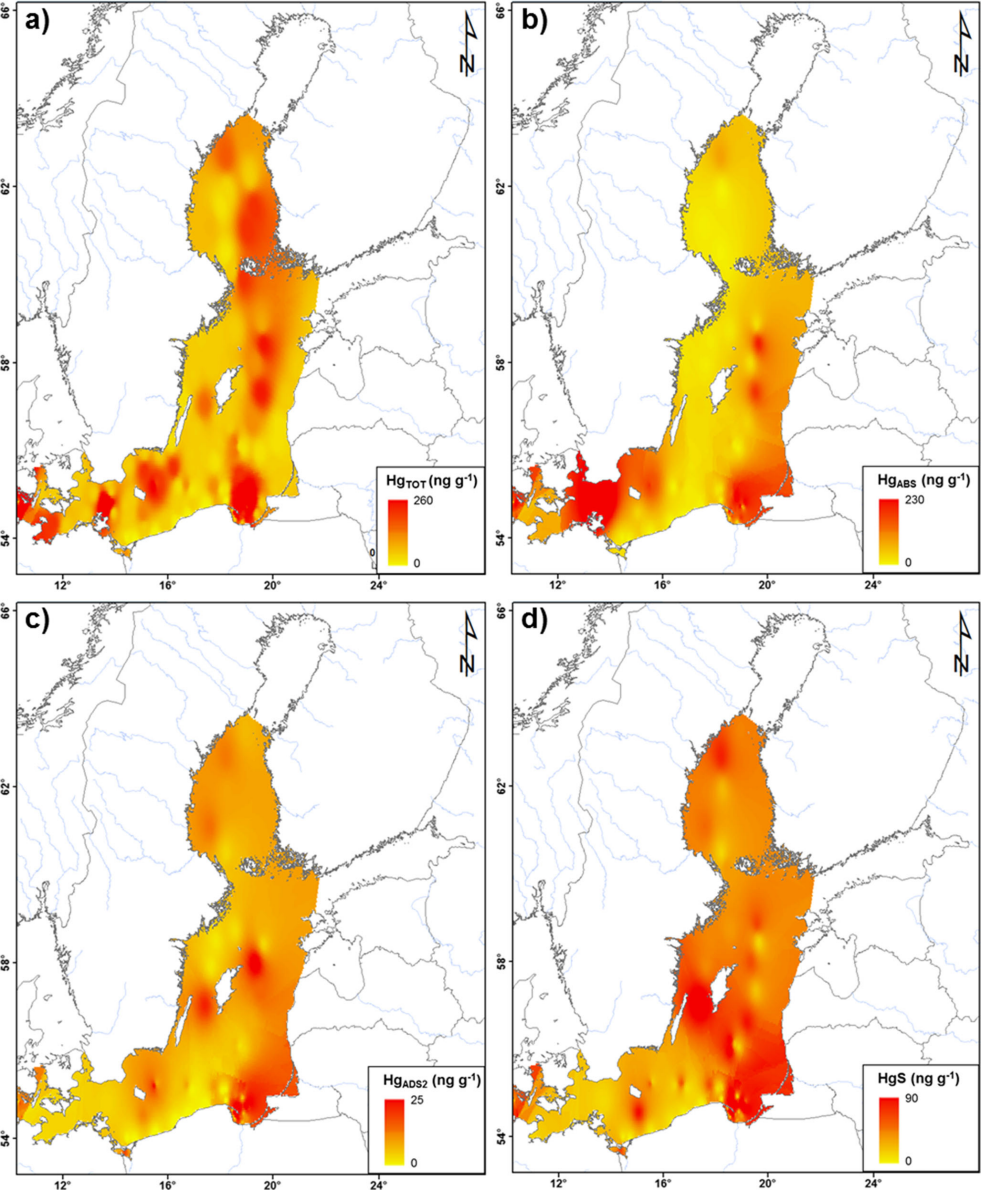
Estimated distribution of total mercury Hg_TOT_ **a** and mercury fractions with the largest percent contribution: Hg_ABS_ **b**, Hg_ADS2_ **c** and HgS **d** concentrations (ng g^−1^) in the surface sediments of the Baltic Sea (interpolated from point data using an inverse distance weighted technique)

Individual regions of the Baltic Sea significantly differed in terms of both Hg_TOT_ concentration and particular Hg fractions (Figs. 3 and 4). The differences in Hg_TOT_ concentration among regions were statistically significant (Kruskal-Wallis test, *p* = 0.001) as was in the case of the contents of 5 Hg forms (Kruskal-Wallis test, *p* < 0.05). Moreover, a statistically significant difference between Hg_TOT_ concentration in sand and soft sediments was found in all of the studied regions (*U* Mann-Whitney test, *p* = 0.000).

#### Belt Sea

In the Belt Sea region, samples of surface sediment were collected at 27 stations, and the concentration of Hg_TOT_ in surface sediments varied within a very wide range from 6.0 to 340.8 ng g^−1^ (median 109.7 ng g^−1^) (Fig. 3a). The wide spread of the measured values resulted from the mosaic-like distribution of sediments in this area (Winterhalter et al. 1981) (Fig. A2). One third of the stations was characterised by sand sediments, in which Hg_TOT_ occurred in the range from 6.0 to 30.8 ng g^−1^, with the median at 10.9 ng g^−1^, which was over 10 times lower than that recorded for soft sediments (median 123.9 ng g^−1^). Elevated values were measured in the Kiel Bay area (Fig. 4a), which is a region subjected to strong human pressure due to one of the largest ports in the Baltic Sea and a shipyard being located there. The Belt Sea area is characterised by high marine traffic, the highest in the Baltic Sea. The western part of Kiel Bay, at the entrance to the Kiel Canal, is called Kolberger Heide and is a very distinctive region due to the intense military activities conducted in this area during and after World War II (Bełdowski et al. 2019). The potential impact of the dumped ammunition is confirmed by the Hg_TOT_ concentrations measured in this area, the highest observed in the Belt Sea region, reaching 340.8 ng g^−1^ (Fig. 3a; A4). This was also visible in the case of Hg_TOT_ concentration in SPM in this region, in both near-bottom and surface water. The concentration of Hg_TOT_ in SPM collected from the surface water layer was 132.6 ng g^−1^, while in samples collected further away from this area, the average concentration was 20.9 ng g^−1^. It was similar in the case of near-bottom waters, where the Hg_TOT_ concentration in SPM in Kiel Bay (the highest Hg_TOT_ concentration recorded in the Belt Sea region) was 56.3 ng g^−1^, which was a value 2 times higher than that obtained in the remaining part of this region. Increased Hg_TOT_ concentrations in the Belt Sea sediments, reaching 159.0 ng g^−1^, were also measured in Mecklenburg Bay (Fig. 4a). Apart from the proximity of the port of Lübeck, this region was for many years influenced by industrial waste material from a metalwork, which used to be dumped in this area until the 1960s. Importantly, the contaminated surface sediments of the Kiel and Mecklenburg bays, which are Hg hot spots, may be to some extent transported to other seabed regions, mainly to the East, via sediment re-suspension caused by strong winds and waves (Kersten et al. 2005; Leipe et al. 2008, 2013).

The dominant Hg fraction in the surface layer of sediments in the Belt Sea region was labile Hg_abs_, which on average constituted 64% (Fig. 3b). This is important given the high Hg_TOT_ concentrations measured in this area. The high content of organic matrix bound Hg could make the sediments in the Belt Sea region an important secondary source of Hg to organisms living in the marine environment (Fig. 4b). The highest average contribution of labile Hg_abs_ (78%) was found in the region with the highest Hg_TOT_ concentrations in the sediments. What is more, SPM was characterised by the dominance of the Hg_ads1_ (73–90%), both in the surface and near bottom layers (Table A1), which might indicate that after being deposited in the sediments, Hg_ads1_ was released back into the water. The high proportion of Hg_abs_ in sediments can presumably be associated with Hg methylation, e.g., through the activity of SRB under anaerobic conditions (median Eh in sand sediments − 188.5 mV; median Eh in soft sediments − 370.2 mV).

#### Arkona Basin

In the Arkona Basin region, samples were collected from 2 research stations with soft sediments (Fig. A2). Hg_TOT_ concentrations ranged from 227.5 to 254.6 ng g^−1^ (median 241.1 ng g^−1^) (Fig. 3a). Equally high Hg_TOT_ results had been obtained in other studies conducted in this area in 1999–2002 (Bełdowski and Pempkowiak 2007) and 2003–2010 (Leipe et al. 2013). The median of Hg_TOT_ concentrations (241.1 ng g^−1^) was almost 10 times higher than the natural background level (Leipe et al. 2013) (Table 2). A very important source of Hg in the Arkona Basin is the Odra river, which supplies SPM through the Pomeranian Bay estuary towards the north (Löffler et al. 2000; Emeis et al. 2002; Pempkowiak et al. 2005). High Hg concentration in surface sediments may be connected to military activities, i.e., release of Hg containing materials from ships or wrecked submarines (Perttilä and Brügmann 1992; Leipe et al. 2008). Despite the reduction of Hg levels in sediments since WWII, this Hg hot spot is still active through Hg diffusion from deeper layers of the sediment.

**Table 2:** Total mercury (Hg_TOT_) concentration (median and range) (ng g^−1^) in the surface layer of soft sediments from different regions of the Baltic Sea in relation to natural geochemical background (NGB)

	Belt Sea	Arkona Basin	Bornholm Basin	Gdańsk Basin	Western Gotland Basin	Eastern Gotland Basin	Bothnian Sea
Hg_TOT_ (ng g^−1^)	123.6 (40.3–340.8)	241.0 (227.5–254.6)	91.7 (50.7–135.8)	138.1 (64.3–263.6)	20.6 (20.3–71.9)	52.2 (8.4–114.2)	46.7 (20.4–78.3)
NGB (ng g^−1^)	ND	25	25	30	20	20	20
% NGB	ND	964	367	461	103	261	234

NGB values were taken from study by Leipe et al. (2013)

ND, no data

The accumulation of Hg-rich organic matter in Arkona Basin sediments is confirmed by the highest contribution of the Hg_abs_ fraction among the studied regions—93.4% on average (Fig. 3b). As was the case with the Belt Sea, this could also have been related to Hg methylation. In surface sediments collected in the Arkona Basin region, the proportion of the stable HgS averaged 1.5% of Hg_TOT_ (Fig. 4) and was many times lower than the value recorded in the same region by Bełdowski and Pempkowiak (2007) in samples taken in the years 1999–2002. The small proportion of the reduced form of Hg in sediments collected in 2016–2017 was most likely the result of inflows from the North Sea, the frequency and intensity of which started to increase in 2014—about 18 months before sampling began. Of particular importance, here was the so-called Major Baltic Inflow recorded in December 2014, whose intensity was defined as very strong (Feistel et al. 2016). With the inflow of oxygenated water to the Arkona Basin, stable HgS oxidised and, as a consequence, most probably became remobilised in the water column. This is confirmed by a decrease of over 20% in Hg_TOT_ concentration in the sediments of the Arkona Basin compared to the values measured in 1999–2002 (305.0 ng g^−1^) (Bełdowski and Pempkowiak 2007). In the case of samples collected in 1999–2002, the high HgS concentration in Arkona Deep sediments, reaching 130 ng g^−1^ (Bełdowski et al. 2009), was associated primarily with the presence of hypoxia and anoxia in the near bottom zone during this period (HELCOM 2010).

#### Bornholm Basin

Hg_TOT_ concentrations in the Bornholm Basin surface sediments (*n* = 15) were found in a wide range from 1.3 to 135.8 ng g^−1^ (median 10.6 ng g^−1^ ) (Fig. 3a). Most stations are located in a region with a sandy bottom (Fig. A2), where Hg_TOT_ concentrations occurred within a range of 1.3 to 18.4 ng g^−1^ with a median of 2.7 ng g^−1^, which was much lower than the median obtained for soft sediments (median 91.7 ng g^−1^). The highest Hg_TOT_ concentrations were recorded in silt sediments in the deep parts of the Bornholm Basin, in the area around the Bornholm Deep (depth > 90 m), where Hg_TOT_ ranged from 91.5 to 135.8 ng g^−1^. Lower concentrations (64.0–70.1 ng g^−1^) were recorded in the years 1999–2002 (Bełdowski and Pempkowiak 2007). The increase in Hg_TOT_ concentration in this region over 10–15 years may suggest the transport of Hg from FSF from the Arkona Basin—a region with high Hg concentration—under the influence of water inflows from the North Sea that occurred in 2014–2016 (one classed as very strong in intensity and several referred to as moderate (Feistel et al. 2016)). Hg_TOT_ concentrations in the Bornholm Basin were almost half as low as those from the Belt Sea and the Arkona Basin (Fig. 3a), which confirms the thesis that most of the pollutants introduced via the Odra river are deposited in the Arkona Basin area (Löffler et al. 2000; Emeis et al. 2002; Pempkowiak et al. 2005). Hg_TOT_ concentrations measured in the Bornholm Basin sediments were more than 3 times higher than the value considered to form the geochemical background for Hg (Leipe et al. 2013) (Table 2). The Bornholm Basin was known as a military dumping site during and after WWII (ammunition and blasting caps containing Hg used as the primary explosive material). The dumped armaments corrode over the years, leading to the release of Hg into the surrounding sediments (Bełdowski et al. 2019). Hg_TOT_ concentration in SPM in the Bornholm Basin region was half as low as that obtained in the case of Kiel Bay. It amounted to 53.6 ng g^−1^ in surface water while in the bottom water, the value was 34.1 ng g^−1^. The Hg form that prevailed in SPM samples (collected from both near-bottom and surface water) was, as in the case of the Belt Sea, Hg_ads1_ (66.0–73.0%) (Table A2).

The predominant Hg fraction in the collected surface sediments was the labile Hg_abs_, which was mainly constituted by Hg bound with organic matter, although its average contribution was lower than 50% (mean 41.9%) (Fig. 3b). Stable HgS (mean 35.0%) had a similar proportion, which was many times higher than that obtained for the Arkona Basin (mean 1.5%). At the 3 deepest stations, located in the Bornholm Deep region, the Hg_abs_ fraction (mean 81.4%) was markedly predominant, and stable HgS was at a level of 8.5%. This may indicate the occurrence of methylation processes under the reductive conditions (median Eh − 152.9 mV). It can also suggest an inflow of Hg-rich organic matter from the Arkona Basin, including that transported with inflows from the North Sea and deposited in the Bornholm Basin. The inflow of fresh organic matter is an important factor shaping the contribution of Hg forms in the Bornholm Basin.

#### Gdańsk Basin

In the samples from the Gdańsk Basin region, a wide range of Hg_TOT_ concentrations in surface sediments was observed: from 1.2 to 263.6 ng g^−1^ (median 121.0 ng g^−1^) (Fig. 3a). The Hg_TOT_ concentrations in soft sediments ranged from 59.8 to 263.6 ng g^−1^, with a median of 137.6 ng g^−1^. In sand sediments, much lower Hg_TOT_ concentrations were recorded, with a concentration range of 1.2 to 25.0 ng g^−1^ (median 7.8 ng g^−1^). The highest Hg_TOT_ concentration (263.6 ng g^−1^) was measured at the station located in the sheltered Puck Bay (Fig. 4a). The bottom morphology, as well as the increased Hg inflow (mainly from anthropogenic sources located on the western shore of the Puck Bay) resulted in increased Hg deposition in this region (Bełdowska et al. 2016; Jędruch et al. 2017; Kwasigroch et al. 2018). Elevated Hg_TOT_ concentrations were also observed in the Gdańsk Deep (depth > 100 m) (Fig. A1). The range of Hg_TOT_ concentrations in surface sediments in this region was from 97.3 to 237.6 ng g^−1^ (median 162.5 ng g^−1^) (Fig. 4a). Similar results in the Gdańsk Deep region were obtained in 1999–2002 (116.0–216.0 ng g^−1^) (Bełdowski and Pempkowiak 2007). That was influenced by the fact that the Gdańsk Deep is a region of deposition and accumulation of matter brought in from the river Vistula (Bełdowski et al. 2009; Saniewska et al. 2014; Jędruch et al. 2015), which is the second largest river flowing into the Baltic. Despite the high Hg_TOT_ concentrations in surface sediments, relatively low Hg concentrations were recorded in surface (48.4–49.0 ng g^−1^) and near-bottom SPM (20.5–28.5 ng g^−1^). The impact of human activity on the Hg level in surface sediments is confirmed by the fact that the values measured in the sediments of the Gdańsk Basin are almost 5 times higher than the natural background of the element in this part of the Baltic (Leipe et al. 2013).

The proportion of individual Hg fractions in surface sediments in the Gdańsk Basin region was shaped in a very similar way to that of the Belt Sea (a region under strong anthropopressure). The dominant form of Hg was labile Hg_abs_, the average proportion of which was 60.8% (Fig. 3b). The distribution of Hg fractions in SPM was also similar in these two areas (Table A2). Hg_ads1_ clearly dominated in SPM in the Gdańsk Basin, both in the surface layer (median 83.8%) and the near-bottom layer (median 73.4%). The contribution of this form was particularly high in the deep regions of the Gdańsk Basin. In shallower areas, on the other hand, SPM was enriched with organic substances originating from increased primary production—in these regions, Hg_abs_ was the predominant form, and its contribution reached 38.8% (Table A2).

A high proportion of HgS in the sediments of the Gdańsk Deep, reaching 50–60% in its southern slope (Fig. 4d), indicates that despite the improvement in oxygen conditions in the Baltic Proper, in the case of the Gdańsk Deep, no significant increase in near-bottom oxygenation was noted (Feistel et al. 2016). It is confirmed by mean Eh at a level of − 101.0 mV, as well as by the results of research by Mohrholz et al. (2015), according to which oxygen concentrations in near-bottom water in the Gdańsk Deep after an inflow still did not exceed 2 ml L^−1^.

#### Gotland Basin

The median of Hg_TOT_ concentrations in the Eastern Gotland Basin region was 52.2 ng g^−1^ (Fig. 3a) which was more than 2 times higher than the natural background (Table 2). However, the concentrations ranged from 7.1 ng g^−1^ in sandy sediments of the Słupsk Furrow (transportation zone) to 114.2 ng g^−1^ in soft sediments of the Gotland Deep (accumulation zone) (Fig. 4a). The median concentration in the Gotland Deep region was 112.5 ng g^−1^ , which is close to that obtained in other studies conducted in this area (Leipe et al. 2013; Bełdowski et al. 2014). The concentration median recorded in the samples from the Western Gotland Basin was two times lower (20.6 ng g^−1^) than in the eastern part of the basin (Fig. 3a). The type of sediment has an additional impact on low concentrations—a large area of the seabed is covered in till, which is characterised by low organic matter content (Fig. A2). This is mainly due to scant surface runoff in this part of the Baltic Sea. SPM introduced from the mainland is deposited mainly in the fjords and fjards (Kyryliuk and Kratzer 2019) on the west coast of the basin (Fig. A1). The difference between the west and east of the Gotland Basin is also reflected in the contribution of individual forms of Hg in the sediments. The eastern part of the basin was dominated by the Hg_abs_ labile form (median 60.8%), while stable HgS (63.2%) predominated in the Western Gotland Basin (Fig. 3b). The near-bottom water collected from the western part of the basin smelled of hydrogen sulphide. Significant oxygen deficiencies were confirmed by low Eh values in the collected sediment (Eh < − 350 mV). The presence of anaerobic conditions and of H_2_S is also indicated by the results of research by Naumann and Nausch (2019) conducted in the same period (May 2017). Equally low Hg_TOT_ levels of 10 ng g^−1^ were measured in till sediments from the Baltic cliffs, where the HgS contribution ranged from 22.0 to 58.1% (Bełdowska et al. 2016; Kwasigroch et al. 2018).

#### Bothnian Sea

The median of Hg concentrations in the Bothnian Sea region was low (25.5 ng g^−1^), similar to that obtained for the Western Gotland Basin (Fig. 3a). In both region,s the seabed is predominantly hard (Fig. A2). The maximum concentration (78.3 ng g^−1^) was recorded at the only station dominated by silty sediments. Despite good aerobic conditions in the near-bottom zone of the basin, the dominant form of Hg was largely reduced HgS (49%) (Fig. 3b). It is likely that this form was introduced into the environment from anthropogenic sources (e.g., from surrounding industrialised regions, including numerous pulp and paper mills and other factories related to the cellulose industry) (Fig. 1) (Sundqvist et al. 2009). It is worth emphasising the high proportion, compared to other regions, of the labile Hg_ads2_ form (25%) (Fig. 3b), i.e., Hg bound with, e.g., iron oxides. This may be due to the fact that numerous ferromanganese concretions are found in the Gulf of Bothnia together with relatively good oxygen conditions (Boström et al. 1982). A significant factor is also the leaching of Fe-rich organic terrestrial matter by rivers, particularly from forest areas or peat bogs, and their subsequent transport to the sea. An additional, secondary source of Fe is the deeper, anaerobic, bottom sediment layers (Gelting et al. 2010; Lenstra et al. 2018).

## Conclusion

The main factor determining the total Hg concentration in surface sediments in the Baltic Sea region was the type of sediment and was associated with the proportion of organic matter and the fine-grained fraction in sediments. In the studied sediments, the influence of modern and historical human activity on Hg accumulation was clearly visible. The highest Hg_TOT_ concentrations in sediments were found in the Kiel Bay, which is a highly industrialised area, and also used to be an area of ammunition dumping following WWII. The elevated concentrations were also recorded in the Arkona Basin region, which was related to inflow of Hg-rich riverine SPM and the morphology of the seabed. The impact of rivers on the elevated Hg_TOT_ concentrations in sediments was also noted in the Gdańsk Basin. The total contribution of labile Hg forms, i.e., Hg associated mainly with halides (Hg_ads1_), Hg in organic connections (Hg_abs_) and Hg in the form of oxide and sulphate (Hg_ads2_), in the Baltic Sea sediments averaged 67 %. This value is higher than the previous estimations, according to which about half of Hg may be remobilised from Baltic sediments. The high proportion of labile Hg forms was most likely the result of water inflows from the North Sea which improved the oxygen conditions in the near-bottom zone of the Baltic Sea and contributed to the oxidation of stable Hg forms in sediments. Importantly, in most regions, the dominant form of Hg was labile Hg_abs_. Stable HgS prevailed only in the Western Gotland Basin, a region with the long-term oxygen deficiency and the presence of H_2_S. Oxygen conditions in the Baltic Sea have been constantly deteriorating since the 1960s (anoxic conditions affect 22% of the Baltic Proper bottom areas and 32% are affected by hypoxia) (Hansson et al. 2018) and therefore may contribute to the ‘retention’ Hg in sediments. On the other hand, North Sea water inflows favour remobilisation of Hg from sediments and its transformation into bioavailable labile forms. They can therefore affect an increase of the load of this element introduced into the trophic chain. This is particularly important as, despite the significant reduction of anthropogenic Hg emission into the Baltic in recent decades (HELCOM 2010; KOBiZE 2019), surface sediments can be an important secondary Hg source in the marine ecosystem. This is especially prevalent in the case of the western Baltic Sea, including the Belt Sea and Arkona Basin, where Hg_TOT_ concentrations in sediments are the highest, while the impact of inflows in this part of the basin is the most intense (Mohrholz et al. 2015). These basins may become veritable hot spots of bioavailable Hg in the Baltic Sea.

## Supplementary information

ESM 1 (PDF 684 kb)

## Data Availability

The datasets used and analysed during the current study are available from the corresponding author on reasonable request.
