# Supplementary material for: Distribution and bioavailability of mercury in the surface sediments of the Baltic Sea
Source: Environ Sci Pollut Res Int. 2021 Mar 6;28(27):35690–708. doi: 10.1007/s11356-021-13023-4 (PMC8277639; doi:10.1007/s11356-021-13023-4)
Supplement: Supplementary file 1 — (PDF 684 kb) [file 11356_2021_13023_MOESM1_ESM.pdf]

SUPPLEMENTARY MATERIAL

to the article *Distribution and bioavailability of mercury in the surface sediments of the Baltic Sea* by Urszula Kwasigroch, Magdalena Bełdowska, Agnieszka Jędruch and Katarzyna Łukawska-Matuszewska (corresponding author: U. Kwasigroch ✉ urszula.kwasigroch@gmail.com)

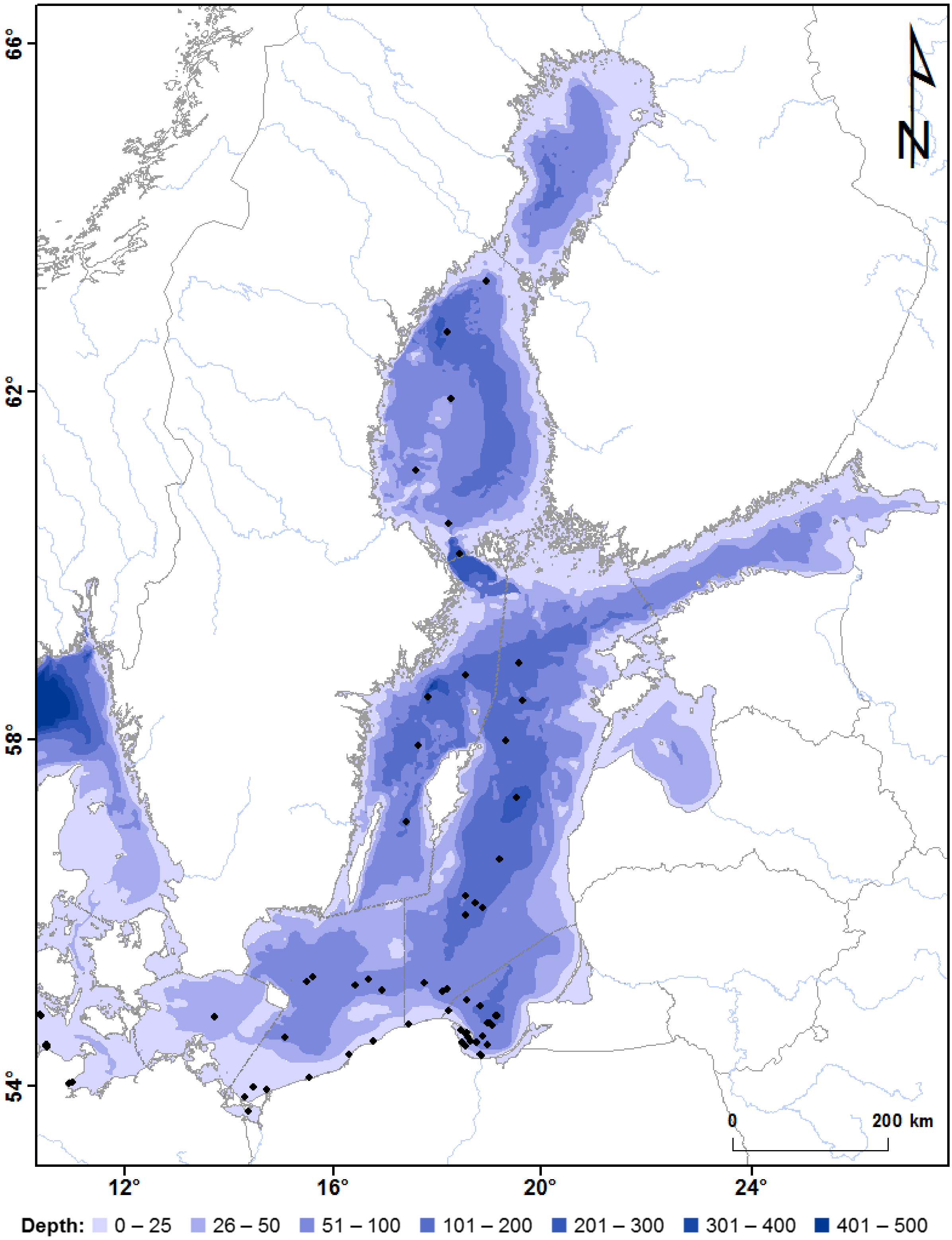

Figure A1 Water depth (m) of the Baltic Sea (digitised from Winterhalter et al., 1981) together with the sampling stations location

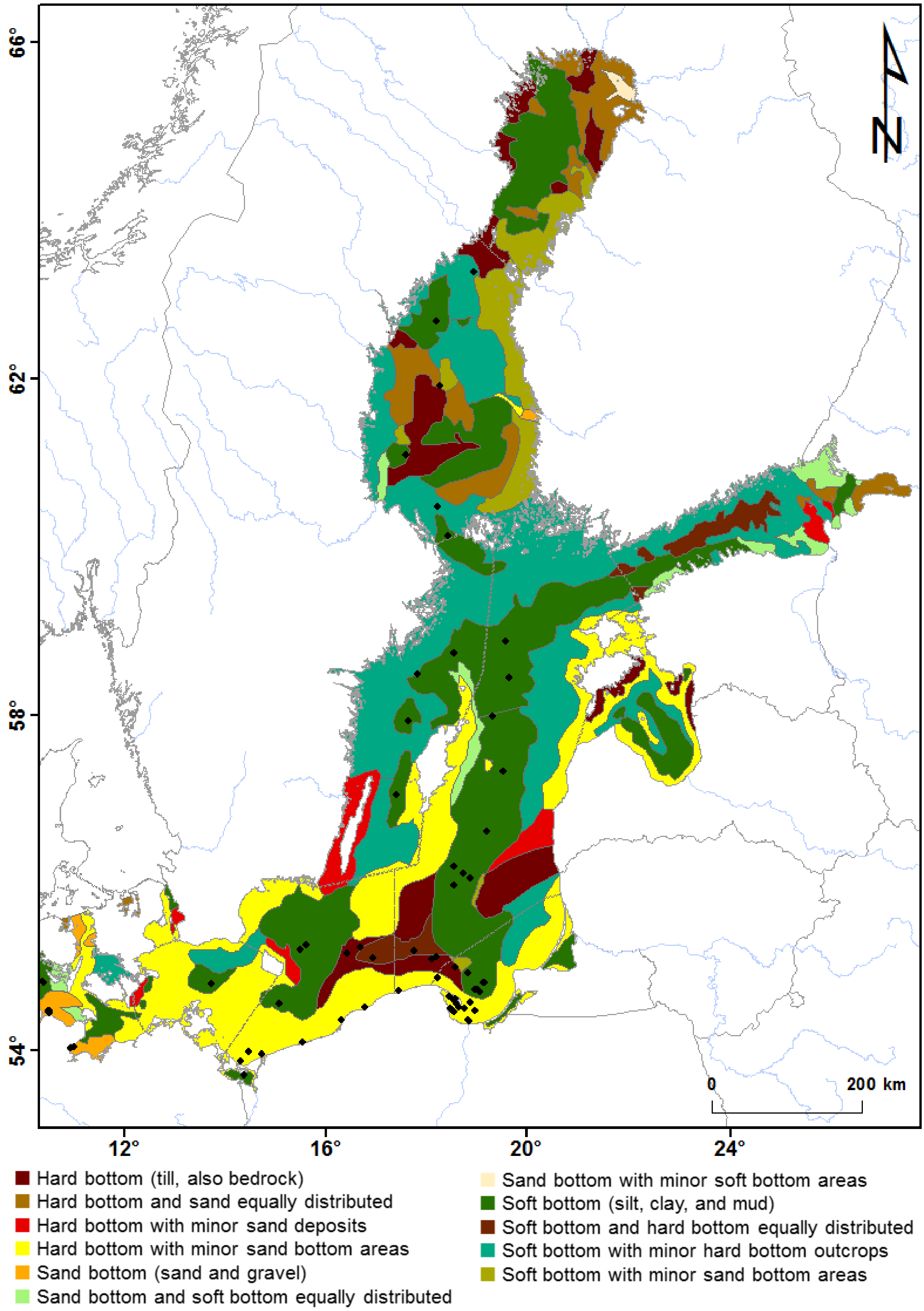

Figure A2 Sediments of the Baltic Sea (digitised from Winterhalter et al., 1981) together with the sampling stations location

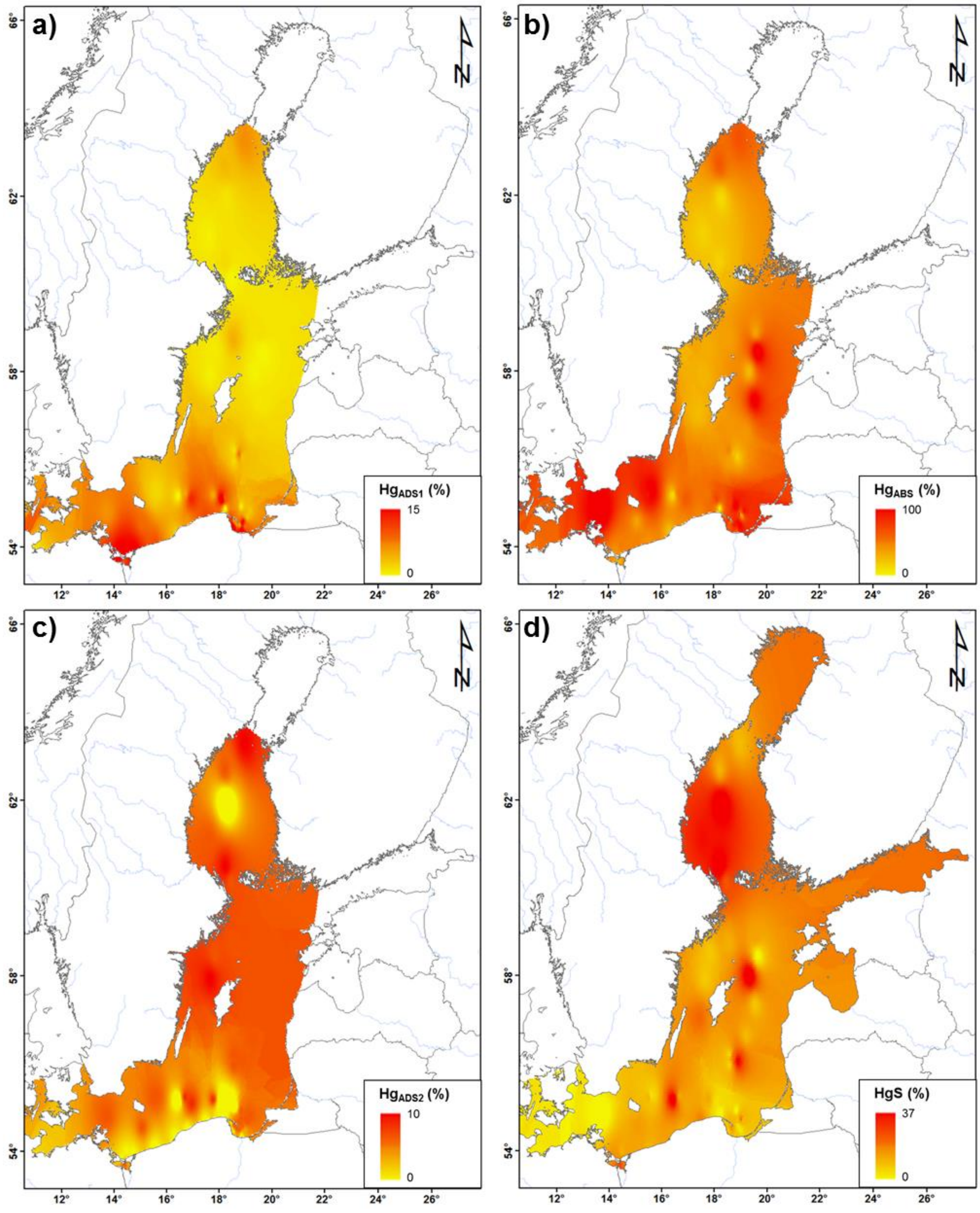

Figure A3 Estimated distribution of (**a**) Hg$_{ADS1}$, (**b**) Hg$_{ABS}$, (**c**) Hg$_{ADS2}$, and (**d**) HgS contribution (%) in the total mercury in the surface sediments of the Baltic Sea (interpolated from point data using an inverse distance weighted technique)

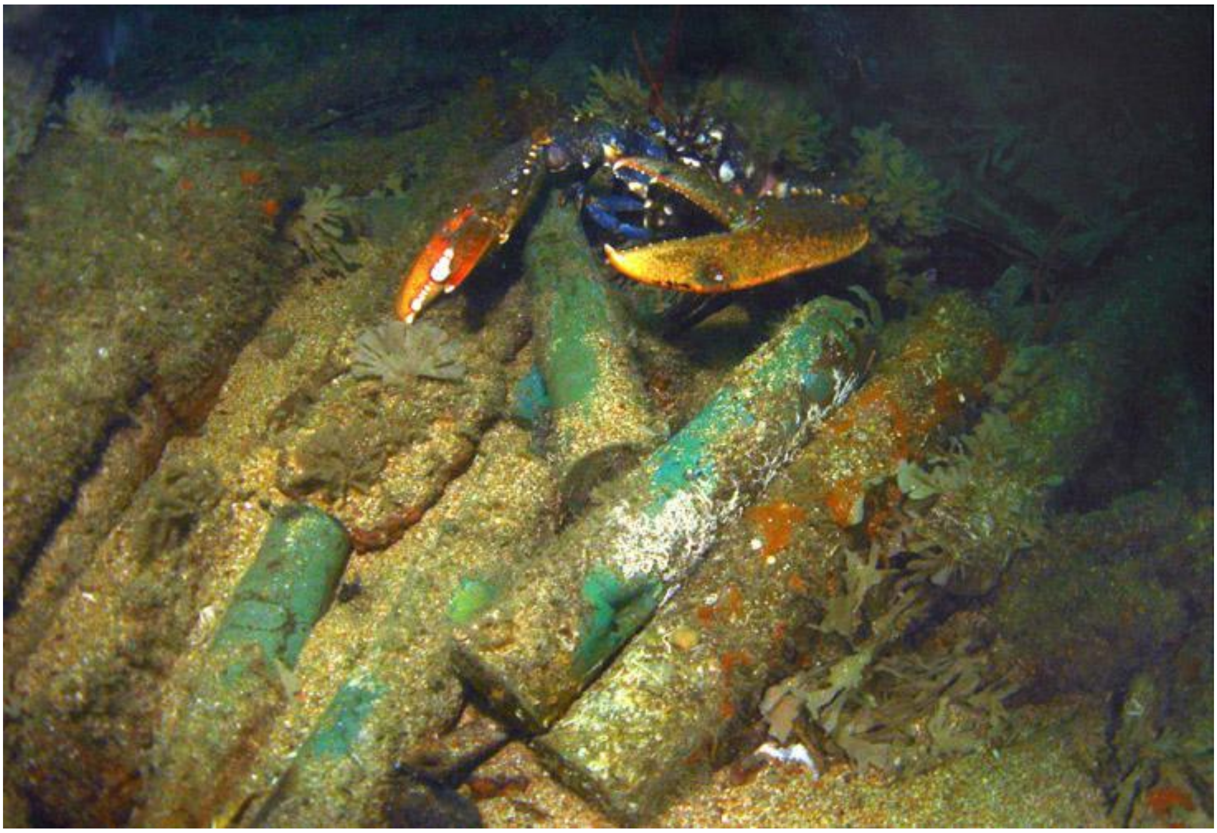

Figure A4 Conventional munitions (such as bombs, grenades, torpedoes and mines) dumped in the sea as a source of Hg to the environment including benthic organisms (ospar.org)

Table A1 Location of the sediment sampling station and the sampling method used

No.	Region	Longitude (E)	Latitude (N)	Water depth (m)	Sampling tool
1	Arkona Basin	13.655583	54.884900	45	van Veen grab
2	Arkona Basin	13.652250	54.883750	45	van Veen grab
3	Belt Sea	10.185917	54.806650	29	van Veen grab
4	Belt Sea	10.188267	54.806283	34	van Veen grab
5	Belt Sea	10.146600	54.824183	33	van Veen grab
6	Belt Sea	10.331233	54.460717	12	van Veen grab
7	Belt Sea	10.313400	54.472917	18	van Veen grab
8	Belt Sea	10.310333	54.476000	18.5	van Veen grab
9	Belt Sea	10.336933	54.453083	6.5	van Veen grab
10	Belt Sea	10.334046	54.457746	7	box corer
11	Belt Sea	10.334041	54.457810	7	box corer
12	Belt Sea	10.330061	54.458956	7	box corer
13	Belt Sea	10.329928	54.458998	7	box corer
14	Belt Sea	10.331839	54.460525	12	box corer
15	Belt Sea	10.310281	54.476062	7	box corer
16	Belt Sea	10.886733	54.067017	18	van Veen grab
17	Belt Sea	10.883067	54.062333	21	van Veen grab
18	Belt Sea	10.824583	54.041517	18	van Veen grab
19	Belt Sea	10.821783	54.046167	17	van Veen grab
20	Belt Sea	10.821750	54.042433	19	van Veen grab
21	Belt Sea	10.821750	54.042433	19	van Veen grab
22	Belt Sea	10.340200	54.454950	10	van Veen grab
23	Belt Sea	10.337333	54.472567	17	van Veen grab
24	Belt Sea	10.317533	54.461850	11	van Veen grab
25	Belt Sea	10.338233	54.463167	15	van Veen grab
26	Belt Sea	10.341583	54.460117	14	van Veen grab
27	Belt Sea	10.340717	54.460117	14	van Veen grab
28	Belt Sea	10.340883	54.460350	14	van Veen grab
29	Belt Sea	10.322750	54.472767	18	van Veen grab
30	Bornholm Basin	16.840967	54.600333	16	van Veen grab
31	Bornholm Basin	16.358367	54.447133	19	van Veen grab
32	Bornholm Basin	15.541383	54.192550	12.5	van Veen grab
33	Bornholm Basin	14.709817	54.044950	10.5	van Veen grab
34	Bornholm Basin	14.364100	53.793950	5	van Veen grab
35	Bornholm Basin	14.287250	53.955600	9	van Veen grab
36	Bornholm Basin	14.449517	54.082217	12.5	van Veen grab
37	Bornholm Basin	15.061217	54.649450	58	van Veen grab
38	Bornholm Basin	15.521533	55.302617	95	van Veen grab
39	Bornholm Basin	17.031100	55.178450	65	van Veen grab
40	Bornholm Basin	16.497700	55.253500	60	van Veen grab
41	Bornholm Basin	17.030067	55.181583	72	box corer
42	Bornholm Basin	16.784300	55.313133	70	box corer
43	Bornholm Basin	15.631417	55.358850	100	van Veen grab
44	Bornholm Basin	15.638850	55.360400	103	van Veen grab
45	Bothnian Sea	19.151183	60.183150	304	van Veen grab
46	Bothnian Sea	19.166550	61.983583	69.5	van Veen grab
47	Bothnian Sea	19.196083	62.759033	174	van Veen grab
48	Bothnian Sea	20.283450	63.317000	98	van Veen grab
49	Bothnian Sea	18.228983	61.184417	70.5	van Veen grab
50	Bothnian Sea	18.931800	60.541217	130	van Veen grab
51	Eastern Gotland Basin	17.561750	54.787083	14	van Veen grab
52	Eastern Gotland Basin	18.250717	55.148183	68	van Veen grab
53	Eastern Gotland Basin	17.905950	55.247817	84	box corer
54	Eastern Gotland Basin	18.819973	56.009467		box corer
55	Eastern Gotland Basin	18.842657	56.243083	100	box corer
56	Eastern Gotland Basin	18.842657	56.243083	100	box corer
57	Eastern Gotland Basin	19.036617	56.147205	128	box corer
58	Eastern Gotland Basin	19.166400	56.082200	122	van Veen grab
59	Eastern Gotland Basin	19.580483	56.633200	140	van Veen grab
60	Eastern Gotland Basin	20.050333	57.333517	244	GEMAX corer

Table A1 Location of the sediment sampling station and the sampling method used

No.	Region	Longitude (E)	Latitude (N)	Water depth (m)	Sampling tool
61	Eastern Gotland Basin	19.900483	58.000400	200	van Veen grab
62	Eastern Gotland Basin	20.334083	58.441167	119	GEMAX corer
63	Eastern Gotland Basin	20.316200	58.884433	157.5	van Veen grab
64	Eastern Gotland Basin	18.367433	55.167733	76	van Veen grab
65	Gdansk Basin	18.963617	54.379017	16	van Veen grab
66	Gdansk Basin	18.770900	54.545850	48	van Veen grab
67	Gdansk Basin	18.751383	55.034667	97	van Veen grab
68	Gdansk Basin	19.328700	54.833883	105	van Veen grab
69	Gdansk Basin	19.169333	54.748550	101	van Veen grab
70	Gdansk Basin	19.231467	54.714000	89	van Veen grab
71	Gdansk Basin	18.698700	54.647483	35	van Veen grab
72	Gdansk Basin	18.669400	54.492033	25	van Veen grab
73	Gdansk Basin	19.137567	54.750650	97	box corer
74	Gdansk Basin	18.702817	54.646183	34	box corer
75	Gdansk Basin	18.691830	54.644200	37	box corer
76	Gdansk Basin	19.113083	54.499567	74	box corer
77	Gdansk Basin	18.598402	54.547908	12	van Veen grab
78	Gdansk Basin	19.111813	54.500030	65	van Veen grab
79	Gdansk Basin	18.999885	54.949642	100	van Veen grab
80	Gdansk Basin	18.957983	54.384917	16	van Veen grab
81	Gdansk Basin	19.027950	54.593000	21	van Veen grab
82	Gdansk Basin	18.891583	54.532817	68	van Veen grab
83	Gdansk Basin	18.698150	54.647633	36	van Veen grab
84	Gdansk Basin	18.732900	54.599817	48	van Veen grab
85	Gdansk Basin	19.319667	54.833250	113	van Veen grab
86	Gdansk Basin	18.591133	54.685592	12	van Veen grab
87	Gdansk Basin	18.360017	54.912717	23	van Veen grab
88	Western Gotland Basin	19.098667	58.784017	133	van Veen grab
89	Western Gotland Basin	18.252267	58.558083	280	van Veen grab
90	Western Gotland Basin	17.998217	57.999767	163	van Veen grab
91	Western Gotland Basin	17.665650	57.117617	106	van Veen grab

Table A2 Concentration of total mercury Hg_{TOT} (median and range) and the share of labile (Hg_{ADS1}, Hg_{ABS}, Hg_{ADS2}) and stable forms (HgS, Hg_{RES}) of Hg in the suspended particulate matter (SPM) from the different regions of the Baltic Sea

		Belt Sea	Bornholm Basin	Gdansk Basin	Western Gotland Basin	Eastern Gotland Basin	Bothnian Sea
Sub-surface	Hg_{TOT} (ng dm^{-3})	0.5 (0.2-0.7)	0.6 (0.2-0.9)	0.4 (0.2-2.1)	0.1	0.2 (0.1-0.3)	0.3
	Hg_{ADS1} (%)	85.3 (80.8-89.9)	75.7 (61.3-86.8)	83.8 (70.9-88.0)	76.7	83.6 (68.1-89.8)	83.8
	Hg_{ABS} (%)	12.1 (7.2-17.1)	19.1 (9.4-35.0)	14.3 (9.5-23.6)	18.9	10.7 (5.4-29.5)	15.2
	Hg_{ADS2} (%)	0.5 (0.5-0.6)	1.0 (0.3-1.8)	0.9 (0.1-7.4)	2.3	1.0 (0.3-3.9)	0.2
	HgS (%)	1.6 (1.5-1.7)	1.8 (1.1-4.9)	1.3 (0.4-2.4)	1.4	1.1 (0.9-1.3)	0.7
	Hg_{RES} (%)	0.4 (0.2-0.7)	0.5 (0.2-1.2)	0.4 (0.0-1.3)	0.7	0.5 (0.2-0.6)	0.2
Near-bottom	Hg_{TOT} (ng dm^{-3})	0.5 (0.4-0.5)	0.3 (0.3-0.4)	0.4 (0.1-0.9)	0.1	0.2 (0.1-0.3)	0.1
	Hg_{ADS1} (%)	75.6 (73.3-77.9)	67.6 (66.0-69.2)	73.4 (41.8-91.1)	70.6 (53.1-88.1)	79.3 (58.5-84.6)	88.6
	Hg_{ABS} (%)	20.5 (17.8-23.1)	26.4 (22.3-30.7)	16.5 (8.3-38.8)	26.9 (9.9-43.8)	18.9 (12.8-36.5)	9.9
	Hg_{ADS2} (%)	1.4 (1.3-1.4)	2.1 (0.6-3.5)	0.9 (0.2-3.3)	0.5 (0.4-0.5)	0.4 (0.2-0.8)	0.6
	HgS (%)	1.9 (1.5-2.3)	2.6 (2.2-3.1)	3.1 (0.4-16.7)	2.0 (1.4-2.6)	1.6 (1.2-4.0)	0.7
	Hg_{RES} (%)	0.7 (0.6-0.8)	1.3 (0.5-2.1)	0.2 (0.1-0.5)	0.1 (0.1-0.1)	0.2 (0.1-0.8)	0.1

Table A3 Spearman's correlation coefficients between parameters analysed in the study (values marked with * are not statistically significant at the *p* level of 0.05)

	Hg_{TOT} (ng g^{-1})	Hg_{ADS1} (%)	Hg_{ABS} (%)	Hg_{ADS2} (%)	HgS (%)	Hg_{RES} (%)	depth (m)	LOI (%)	FSF (%)	Eh (mV)
Hg_{TOT} (ng g^{-1})		-0.33	0.49	-0.30	-0.38	-0.80	0.12*	0.71	0.87	-0.64
Hg_{ADS1} (%)	-0.33		0.07*	-0.11*	-0.15*	0.26	-0.55	-0.24*	-0.38	0.50
Hg_{ABS} (%)	0.49	0.07*		-0.80	-0.94	-0.64	-0.17*	0.26*	0.13*	-0.39
Hg_{ADS2} (%)	-0.30	-0.11*	-0.80		0.67	0.59	0.24	-0.13*	0.06*	0.41
HgS (%)	-0.38	-0.15*	-0.94	0.67		0.49	0.21*	-0.14*	-0.01*	0.26*
Hg_{RES} (%)	-0.80	0.26	-0.64	0.59	0.49		-0.16*	-0.60	-0.53	0.75
depth (m)	0.12*	-0.55	-0.17*	0.24	0.21*	-0.16*		0.38	0.48	-0.48
LOI (%)	0.71	-0.24*	0.26*	-0.13*	-0.14*	-0.60	0.38		0.76	-0.81
FSF (%)	0.87	-0.38	0.13*	0.06*	-0.01*	-0.53	0.48	0.76		-0.50
Eh (mV)	-0.64	0.50	-0.39	0.41	0.26*	0.75	-0.48	-0.81	-0.50	
